# iRhom2 Mutation Leads to Aberrant Hair Follicle Differentiation in Mice

**DOI:** 10.1371/journal.pone.0115114

**Published:** 2014-12-29

**Authors:** Yang Leilei, Liu Bing, Li Yang, Wang Shaoxia, Xu Yuan, Wang Dongping, Ye Huahu, Shang Shichen, Zhang Guangzhou, Peng Ruiyun, Zeng Lin, Li Wenlong

**Affiliations:** 1 Beijing Institute of Radiation Medicine, Beijing 100850, China; 2 Institute of JingFeng Medical Laboratory Animal, Beijing 100071, China; University of Tennessee, United States of America

## Abstract

iRhom1 and iRhom2 are inactive homologues of rhomboid intramembrane serine proteases lacking essential catalytic residues, which are necessary for the maturation of TNFα-converting enzyme (TACE). In addition, iRhoms regulate epidermal growth factor family secretion. The functional significance of iRhom2 during mammalian development is largely unclear. We have identified a spontaneous single gene deletion mutation of *iRhom2* in *Uncv* mice. The *iRhom2^Uncv/Uncv^* mice exhibit hairless phenotype in a BALB/c genetic background. In this study, we observed dysplasia hair follicles in *iRhom2^Uncv/Uncv^* mice from postnatal day 3. Further examination found decreased hair matrix proliferation and aberrant hair shaft and inner root sheath differentiation in *iRhom2^Uncv/Uncv^* mutant hair follicles. iRhom2 is required for the maturation of TACE. Our data demonstrate that iRhom2*^Uncv^* cannot induce the maturation of TACE in vitro and the level of mature TACE is also significantly reduced in the skin of *iRhom2^Uncv/Uncv^* mice. The activation of Notch1, a substrate of TACE, is disturbed, associated with dramatically down-regulation of Lef1 in *iRhom2^Uncv/Uncv^* hair follicle matrix. This study identifies iRhom2 as a novel regulator of hair shaft and inner root sheath differentiation.

## Introduction

iRhom1 and iRhom2, which are inactive homologues of the rhomboid intramembrane serine proteases that lack the essential catalytic residues [1]. iRhom1 and iRhom2 regulate the secretion of the epidermal growth factor (EGF) family by endoplasmic reticulum-associated degradation [2]. iRhom2 is required for the release of tumor necrosis factor α (TNFα) in macrophages by controlling the maturation of TNFα-converting enzyme (TACE, also called ADAM17) [3]–[5]. Moreover, iRhom2 controls the activation and substrate selectivity of TACE-dependent shedding events [6]. Dominant mutations of iRhom2 is the cause of human tylosis esophageal cancer [7], [8]. In addition, iRhom2 plays an important role in inflammatory arthritis [9]. *iRhom2* knockout mice could survive in a lethal lipopolysaccharide dose [5]. However, the functional significance of iRhom2 during mammalian skin development is unclear.

In the developing hair follicle, signals from adjacent mesenchymal dermal papilla cells instruct the overlying epithelium to form hair placodes [10], [11]. The placode proliferates to form a larger bulb (matrix) and further differentiates into a central hair shaft consisting of the medulla, cortex and hair shaft cuticle surrounded by the inner root sheath (IRS), which consists of the inner root sheath cuticle and Huxley's and Henle's layers. The outer root sheath (ORS) is outside the IRS, is contiguous with the interfollicular epidermis and contains a reservoir of quiescent SCs that are known as the bulge. The bone morphogenetic protein (BMP), Notch and Wnt/β-catenin signaling pathways allow the normal differentiation of matrix cells into the hair shaft and the IRS envelope [12]–[15]. Gata3 is expressed in IRS, the Gata3 mutant mice generate primary IRS defects, which lead to alterations in the shaft [16], [17]. Foxn1 and Hoxc13, which are important hair shaft gene regulators, both of which cause hair defects when mutated [18], [19].

Here, we report a role for iRhom2 in mouse skin development. In a BALB/c genetic background, homozygous *uncovered* (*Uncv*, MGI: 1261908) mice have a hairless phenotype [20], [21]. We identified a spontaneous non-frameshift deletion mutation in the N-terminal cytoplasmic domain of of *iRhom2* (*iRhom2^Uncv^*) in *Uncv* mice by sequence capture array and sequencing platform. iRhom2*^Uncv^* could not induce the maturation of TACE, and dcresease the level of NICD and Lef1. This study suggests that iRhom2 regulates hair shaft and IRS differentiation by specifically modulating Notch1 and Wnt signaling pathway which maybe mediated by TACE.

## Results

### Pattern of *iRhom2* expression in the mouse

In a BALB/c genetic background, homozygous *Uncv* mice are hairless; heterozygous *Uncv* mice have a sparse hair coat (Figure A in S1 Fig.). A previous study demonstrated that the *Uncv* mouse hair abnormalities were linked to a single autosomal gene mutation and incomplete dominant inheritance [20]. *Uncv* mice with heterozygous parents exhibited the expected Mendelian ratio (wild-type:heterozygous:homozygous  = 105∶191∶103). These results show that *Uncv/Uncv* mice only have a single gene mutation. Using a genetic linkage analysis of *Uncv* mice, the mutated gene was mapped to a region between markers D11mit338 and D11mit337 on mouse chromosome 11 [20], [22]. We identified a 309 bp spontaneous non-frameshift deletion mutation in the N-terminal cytoplasmic domain of *iRhom2* (*iRhom2^Uncv^*) in *Uncv* mice by sequence capture array and sequencing platform (Figure B in S1 Fig.). PCR analysis showed that each hairless mouse's genotype is iRhom2*^Uncv/Uncv^*, each sparse mouse's genotype is iRhom2*^Uncv/+^*. The hair phenotype of mouse is depended on the dose of *iRhom2*. All the results indicated that *iRhom2* mutation leads to the hairless in mice.

**Figure 1:**
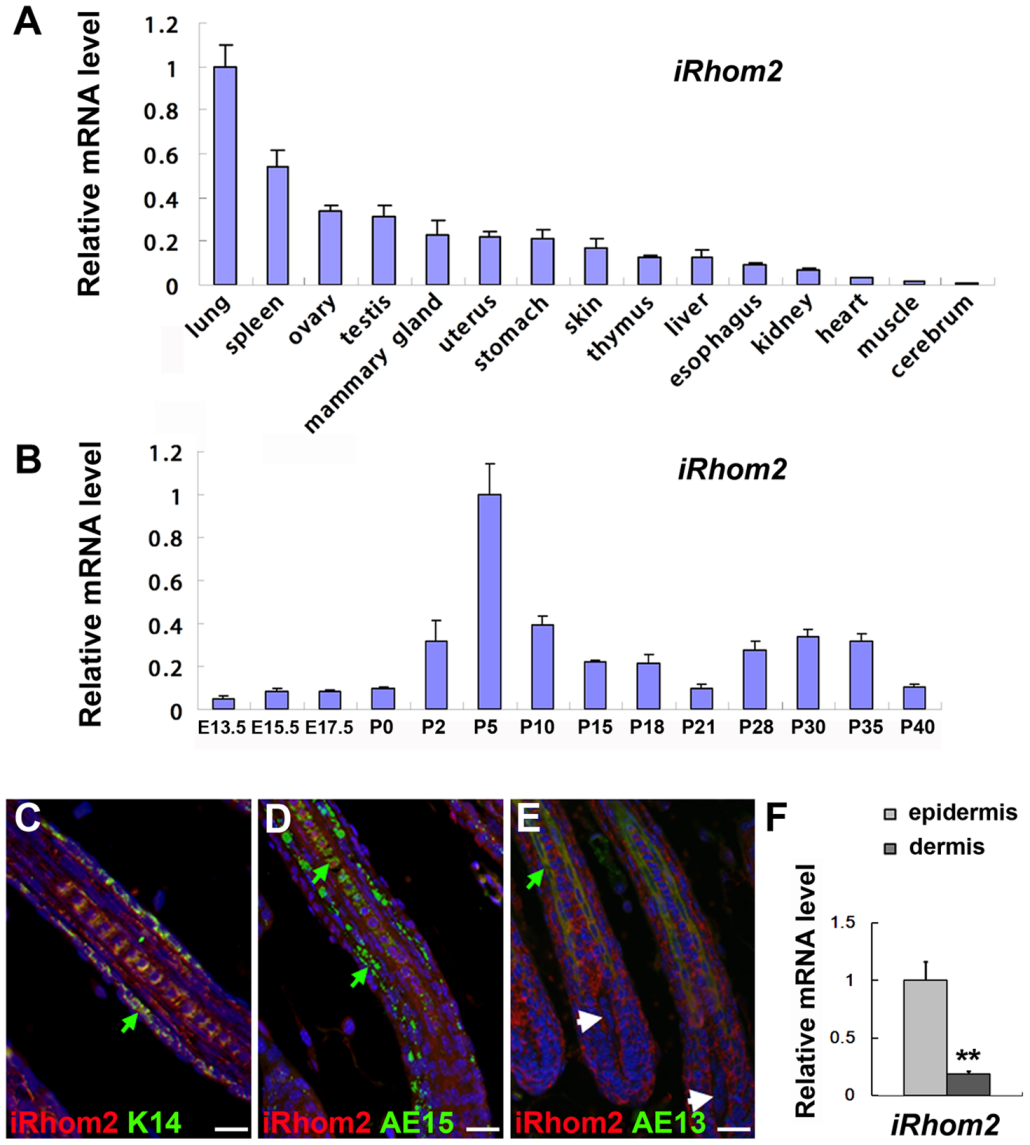
Pattern of *iRhom2* expression in mice. (A) Real-time PCR analysis of *iRhom2* expression in 2-month-old mice. (B) Real-time PCR analysis of *iRhom2* expression during development in the mouse dorsal skin. (C–E) Immunofluorescence staining of iRhom2, K14, AE15 and AE13 in the dorsal skin of wild-type mice at P9. (F) Real-time PCR analysis of *iRhom2* mRNA expression in the epidermis and dermis of wild-type mice at P5. E: embryonic day; P: postnatal day. Green arrow in C indicates the ORS, green arrow in D indicates the IRS and medulla of the hair shaft, green arrow in E indicates the hair shaft cuticle and cortex keratins. White arrow in E indicates the dermal papilla. Scale bars: (C–E), 25 µm.

The pattern of *iRhom2* mRNA expression was analyzed in 8-week-old mice. *iRhom2* was expressed at high levels in the lung and spleen and at moderate levels in the skin (Fig. 1A). Next, we examined the expression of *iRhom2* mRNA during skin development. *iRhom2* was found to be specifically expressed at high levels on postnatal days (P) 2–15 and P28–35 (Fig. 1B), which corresponds well with the hair follicle growth phase (anagen). The expression of *iRhom2* was detected at much lower levels in the hair follicle morphogenesis stage [embryonic day (E) 13.5-P0] and telogen stage (P21) (Fig. 1B). The cyclic expression pattern of *iRhom2* implies that it maybe play a role in the progression of anagen in hair follicles. The strong ubiquitous cellular expression of iRhom2 is detected in the hair follicles (Fig. 1C–E and Figure B in S2 Fig.). Double immunofluorescence staining demonstrated that iRhom2 and K14 (marker for the ORS), AE13 (marker for the hair shaft cuticle and cortex keratins) or AE15 (marker for the the IRS and medulla of the hair shaft) were colocalized in the hair follicles (Fig. 1C–E). iRhom2 was not expressed in dermal papilla cells (Fig. 1E). Moreover, iRhom2 was also expressed in basal layer of epidermis (Figure C in S2 Fig.). There is a non-frameshift deletion mutation of iRhom2 in *Uncv* mice, the antibody we used could not discriminate iRhom2 and iRhom2*^Uncv^*, so iRhom2 expression could still be detected in *iRhom2^Uncv/Uncv^* mice (Figure E in S2 Fig.). However, we performed real-time PCR analysis of *iRhom2* and *iRhom2^#^* (The PCR primers were located in the deletion region of *iRhom2^Uncv^*) in wild type and *iRhom2^Uncv/Uncv^* mice, the results showed that *iRhom2^#^* could not be detected in *iRhom2^Uncv/Uncv^* mice (Figure A in S2 Fig.). To confirm whether *iRhom2* was expressed in the dermis, the expression of *iRhom2* was separately assessed in the epidermis and dermis at P5 using real-time PCR. The results indicated that the dermis expresses very little *iRhom2* (Fig. 1F).

### Aberrant hair shaft and inner root sheath differentiation in *iRhom2^Uncv/Uncv^* mice

**Figure 2:**
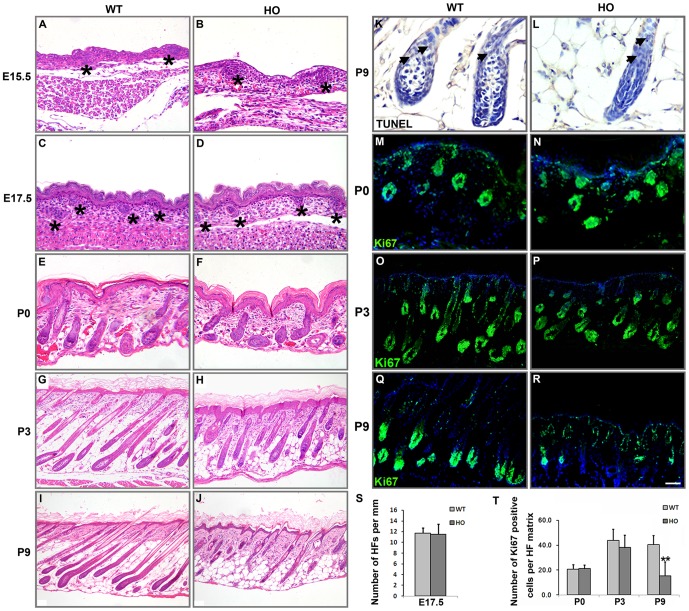
Dysplasia hair follicles in *iRhom2^Uncv/Uncv^* mice. (A–J) Histology of the dorsal skin from wild-type and *iRhom2^Uncv/Uncv^* mice at E15.5, E17.5, P0, P3 and P9, respectively. (K, L) TUNEL staining in the dorsal skin of wild-type and *iRhom2^Uncv/Uncv^* mice at P9. (M–R) Immunofluorescence staining of Ki67 in the dorsal skin of wild-type and *iRhom2^Uncv/Uncv^* mice at P0, P3 and P9, respectively. (S) Number of hair follicles per mm at E17.5; n = 6. (T) Number of Ki67-positive cells per hair follicle matrix at P0, P3 and P9; n = 8, **P<0.01. WT, wildtype; HO, homozygous. Stars indicate the hair placodes. Scale bars: (A–F, M, N), 25 µm; (K, L), 12.5 µm; (G, H, O–R), 50 µm; (I–J), 100 µm.

In mice, the development of the primary hair follicles is initiated at approximately E13 and extends to P16 [10]. To determine whether the deletion mutation in *iRhom2* affects hair follicle morphogenesis, we examined the dorsal skin histology of *iRhom2^Uncv/Uncv^* and wild-type mice at E15.5, E17.5, P0, P3 and P9. At hair follicle morphogenesis stage (E15.5, E17.5 and P0), the follicles of *iRhom2^Uncv/Uncv^* were histologically similar to wild-type mice (Fig. 2A–F). Skin follicle density was not significantly different in *iRhom2^Uncv/Uncv^* mice compared to the wild-type at E17.5 (12±2 compared to 12±1 follicles per mm, respectively; *P* = 0.543, *n* = 8, Fig. 2S). At P3, the differentiating stage, *iRhom2^Uncv/Uncv^* mice showed slightly shorter hair follicles (Fig. 2G and H). However, the skin of *iRhom2^Uncv/Uncv^* mice displayed striking defects in hair follicle morphology at later postnatal stages. By P9, the hair follicles of wild-type mice were in mid-anagen become fully differentiated with large hair bulbs that had descended deep into the fat layer of the skin. In contrast, the hair follicles of *iRhom2^Uncv/Uncv^* mice were smaller and misshapen, and the majority of follicles failed to produce hair shafts (Fig. 2I and J). Few apoptotic cells were detected by TUNEL staining in wildtype and *iRhom2^Uncv/Uncv^* hair follicle matrix of P9 (2.94±1.12 vs. 3.49±1.49, *P = *0.42, *n* = 8, Fig. 2K and L). At P18, hair follicles of both wildtype and mutant mice entered catagen [23] (Figure A and B in S3 Fig.), and subsequently entered telogen stage at P22. At the telogen stage, the hair follicles shortened and condensed in the dermis (Figure C and D in S3 Fig.). At P32, the second anagen, both wildtype and mutant hair follicles elongated and reentered subcutis, however, hair follicles of *iRhom2^Uncv/Uncv^* mice were still misshapen compared with the wildtye mice (Figure E and F in S3 Fig.). Thus the *iRhom2^Uncv/Uncv^* hair follicle cycled normally and the marked shrinkage of the hair follicle matrix in mutant mice is not due to premature catagen development.

During follicle maturation, hair matrix keratinocytes rapidly propagate and differentiate, forming columns of cells that become the hair shaft and IRS [17]. To investigate hair follicular proliferation in *iRhom2^Uncv/Uncv^* mice, we performed Ki67 immunofluorescence staining. In normal hair follicles, a large number of Ki67-positive cells was concentrated in the hair matrix at P0 and P3. A similar number of Ki67-positive cells was observed in *iRhom2^Uncv/Uncv^* mouse follicles at P0 (21.13±2.38 in wild-type follicles compared to 20.75±3.19 in mutant follicles; *P = *0.82, *n* = 8, Fig. 2M, N and T) and P3 (43.88±9.03 in wild-type follicles compared to 38.13±8.72 in mutant follicles; *P = *0.22, *n* = 8, Fig. 2O, P and T). However, compared to P9 wild-type mice, the number of Ki67-positive cells was significantly lower in *iRhom2^Uncv/Uncv^* mice (40.50±7.39 in wild-type follicles compared to 15.38±11.45 in mutant follicles, *P = *1.31E-4, *n* = 8, Fig. 2Q, R and T).

**Figure 3:**
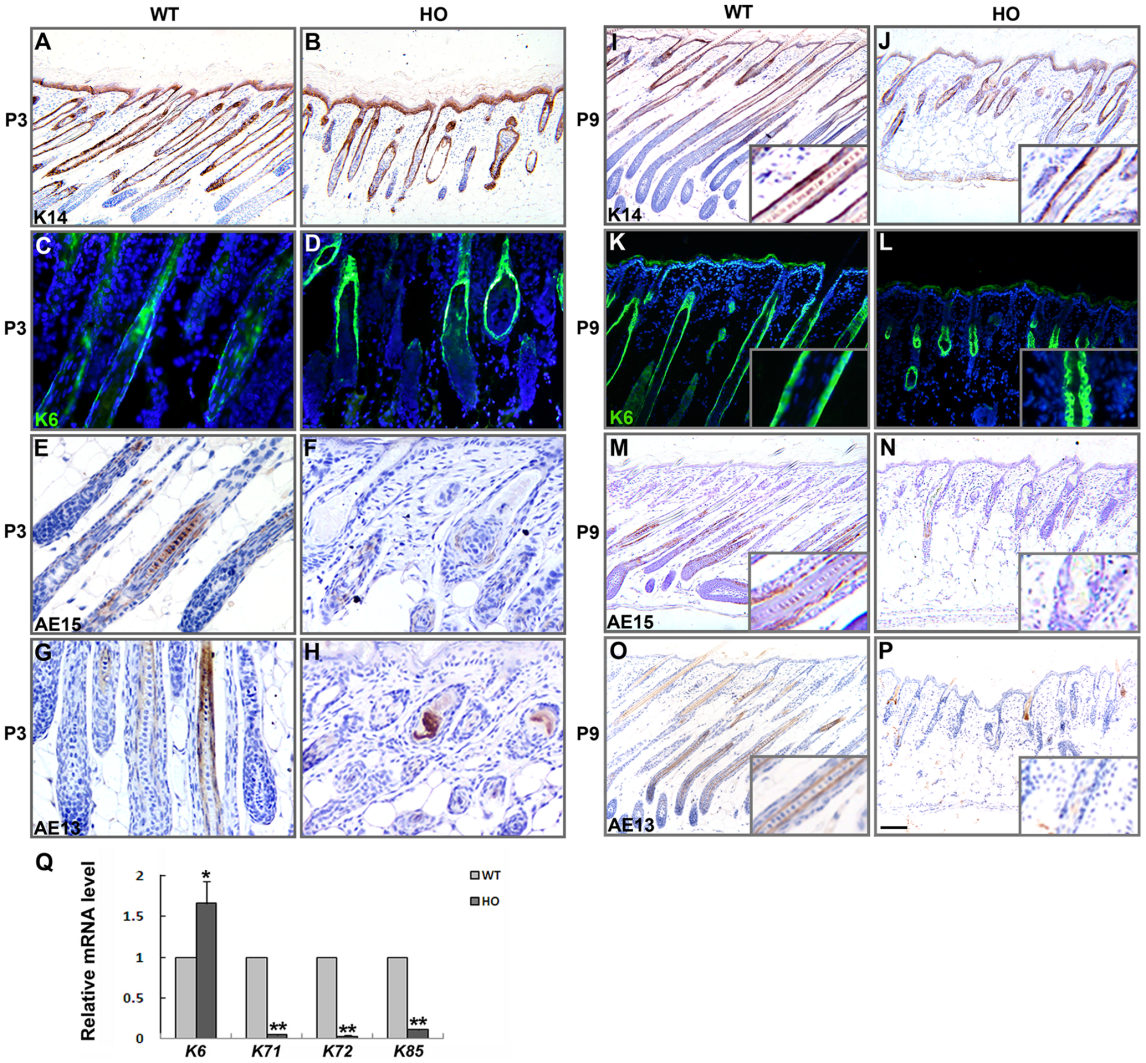
Aberrant hair shaft and inner root sheath differentiation in *iRhom2^Uncv/Uncv^* mice. (A–P) Immunohistochemistry for K14, K6, AE15, AE13 in the dorsal skin of P3 and P9 wild-type and *iRhom2^Uncv/Uncv^* mice. (Q) Real-time PCR analysis of *K6*, *K71*, *K72* and *K85* mRNA expression in the dorsal skin of wild-type and *iRhom2^Uncv/Uncv^* mice at P9. n = 3, *P<0.05, **P<0.01. Scale bars: (A, B, K, L), 50 µm; (C–H), 25 µm. (I, J, M–P), 100 µm.

Next, we examined the expression of several markers of differentiation in the hair follicle at P3 and P9. The ORS expresses keratin (K) 14. In *iRhom2^Uncv/Uncv^* mouse follicles, K14 was expressed at normal levels (Fig. 3A, B, I and J). K6 was expressed in the companion layer of the hair follicle [24]. The expression of K6 was increased in *iRhom2^Uncv/Uncv^* mouse hair follicles (Fig. 3C, D, K and L). In contrast, AE15, which is normally expressed in the IRS and medulla of the hair shaft [25], was markedly reduced in the majority of *iRhom2^Uncv/Uncv^* mouse follicles and was absent from the most distorted follicles, indicating defective IRS differentiation and an absence of the hair shaft medulla (Fig. 3E, F, M and N). AE13, a specific marker for the hair shaft cuticle and cortex keratins [26], was absent in the majority of *iRhom2^Uncv/Uncv^* mouse follicles (Fig. 3G, H, O and P). We confirmed these results by real-time PCR, and the results showed that the expression of IRS markers (*K71* and *K72*) and the hair shaft marker (*K85*) was significantly decreased in *iRhom2^Uncv/Uncv^* mouse follicles and that the expression of *K6* was increased (Fig. 3Q). These results indicate that the hair matrix cells failed to differentiate toward the hair shaft and the IRS in *iRhom2^Uncv/Uncv^* mouse follicles, suggesting a crucial role for iRhom2 in hair follicle differentiation.

### The iRhom2*^Uncv^* mutant protein cannot induce the maturation of TACE

**Figure 4:**
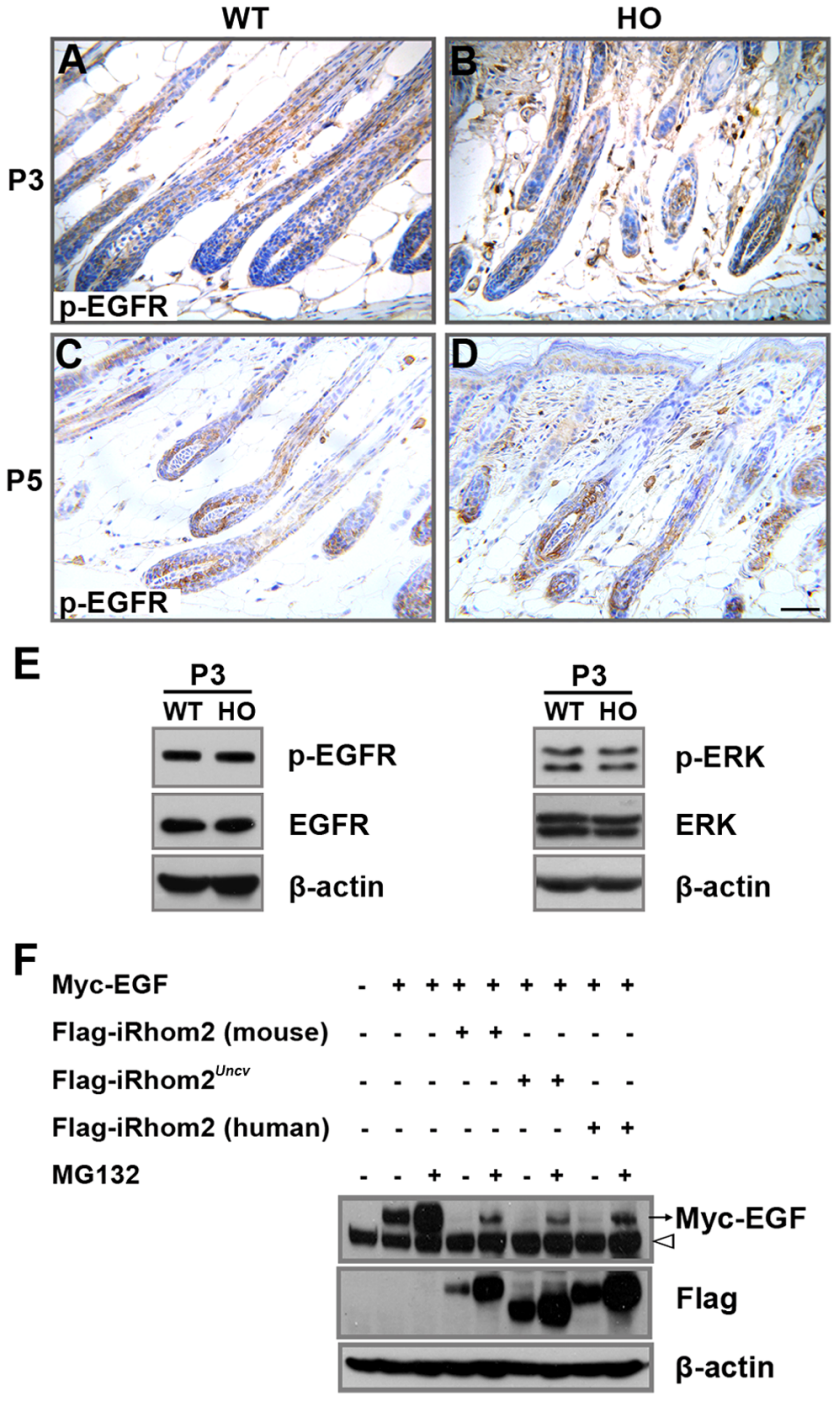
The expression of phospho-EGFR is not affected during follicle differetiation in the *iRhom2^Uncv^* mutation. (A–D) Immunohistochemistry for phospho-EGFR in the dorsal skin of P3 and P5 wild-type and *iRhom2^Uncv/Uncv^* mice. (E) Western blot analysis of phospho-EGFR and phospho-ERK expression in the dorsal skin of P3 wild-type and *iRhom2^Uncv/Uncv^* mice. (F) 293T cells were transfected with the indicated constructs, and the cell lysates were then probed with anti-Flag, anti-Myc and anti-β-actin antibodies (white arrowhead is a non-specific band). Transfected cells were treated with 10 µM MG132 for 12 hours. Scale bars: (A–D), 25 µm.

iRhoms inhibits secretion of EGF family ligands by inducing degradation of epidermal growth factor receptor (EGFR) ligands in mammalian cells [2]. Therefore, we analyzed the expression of phospho-EGFR in P3 and P5 mouse skin. The expression of phospho-EGFR was similar in the *iRhom2^Uncv/Uncv^* and the wild-type hair follicles (Fig. 4A–E), so was the expression of phospho-ERK (Fig. 4E). These data indicated that during hair follicle maturation the expression of phospho-EGFR and phospho-ERK were not altered in *iRhom2^Uncv/Uncv^* mice. Furthermore, an in vitro EGF degradation assay showed that both wild-type iRhom2 and iRhom2*^Uncv^* could promote EGF degradation (Fig. 4F). This degradation is dependent on the proteasome; the proteasome inhibitor MG132 could partly restore the expression of EGF (Fig. 4F). Therefore, the deleted region in the iRhom2*^Uncv^* is not necessary for the iRhom2-mediated degradation of EGF.

**Figure 5:**
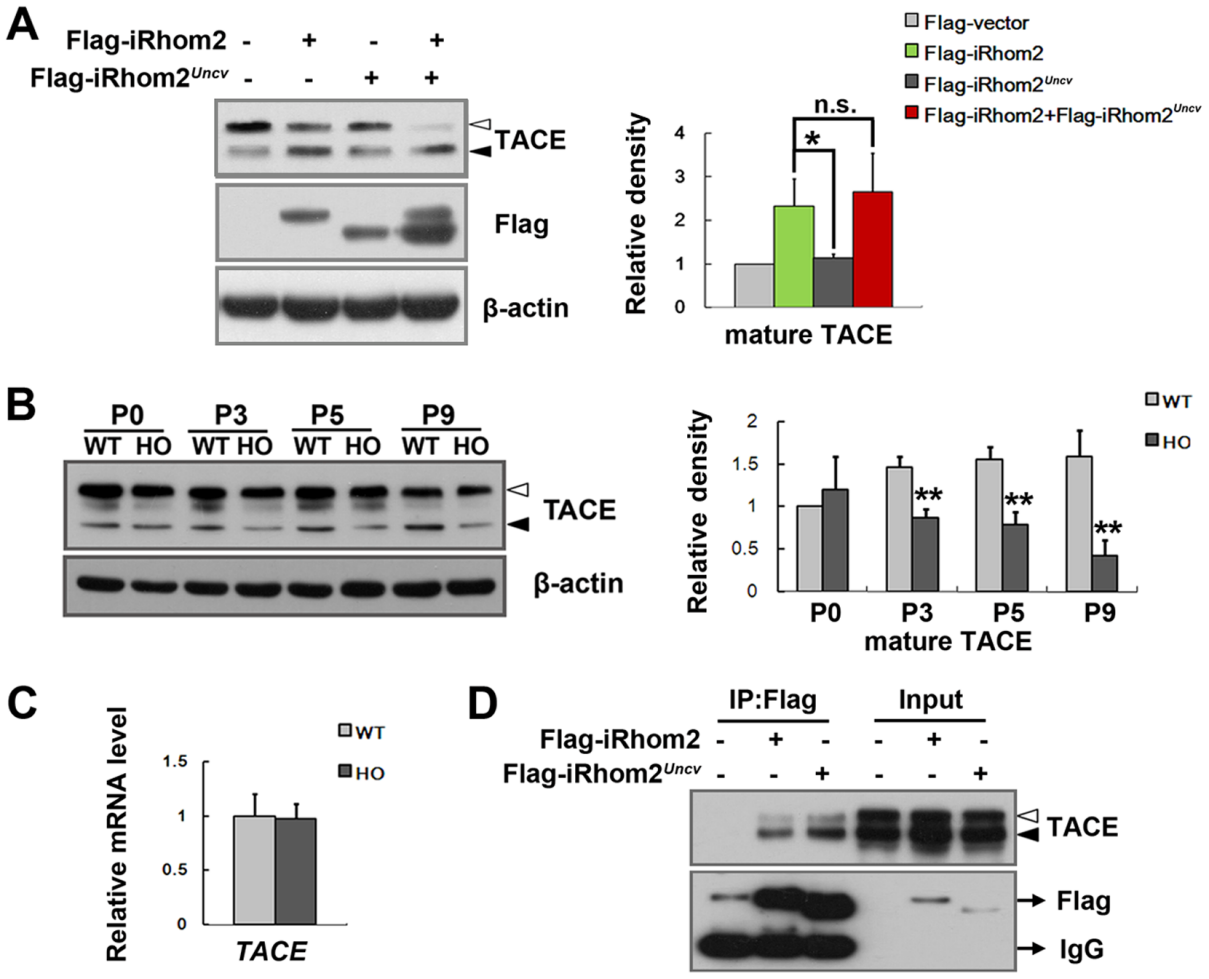
The iRhom2*^Uncv^* mutant protein cannot induce the maturation of TACE. (A) 293T cells were transfected with the indicated constructs. Cell lysates were probed with anti-TACE antibody (immature, white arrowhead; mature, black arrowhead). The histogram shows the relative density of the maturation of TACE. n = 3, *P<0.05. (B) Western blot analysis of TACE expression in the dorsal skin of wild-type and *iRhom2^Uncv/Uncv^* mice (immature, white arrowhead; mature, black arrowhead). The histogram shows the relative density of the maturation of TACE. n = 3, **P<0.01. (C) Real-time PCR analysis of *TACE* mRNA expression in the dorsal skin of wild-type and *iRhom2^Uncv/Uncv^* mice at P5. n = 3. (D) 293T cells were transfected with the indicated constructs. Cell lysates were immunoprecipitated using an anti-Flag antibody, and the immunoprecipitates were probed with an anti-TACE antibody.

It was reported that iRhom2 interacts with TACE and promotes its maturation in macrophages [3]. Immunoprecipitation of iRhom2-overexpressing 293T cells followed by immunoblotting revealed that iRhom2*^Uncv^* did not affect it's interaction with TACE (Fig. 5D). We expressed wild-type iRhom2 and *iRhom2^Uncv^* in 293T cells and assayed its effect on the maturation of TACE. Consistent with previous reports [3], the overexpression of wild-type iRhom2 caused the excessive maturation of TACE; however, exogenous iRhom2*^Uncv^* could not induce the maturation of TACE compared with the empty vector (Fig. 5A). Moreover, exogenous iRhom2*^Uncv^* does not significantly affect the maturation of TACE that is induced by exogenous wild-type iRhom2 (Fig. 5A). In western blots of *iRhom2^Uncv/Uncv^* mouse skin, mature TACE was significantly reduced at P3, P5 and P9 (Fig. 5B). The mRNA of *TACE* was not significantly different between the *iRhom2^Uncv/Uncv^* and wild-type mice at P5 (Fig. 5C). Double immunofluorescence staining revealed that TACE and iRhom2 were colocalized in the hair follicles of wild-type and *iRhom2^Uncv/Uncv^* mice (Figure D and E in S2 Fig.). These results indicate that iRhom2*^Uncv^* cannot induce the maturation of TACE in vitro or in vivo.

### Expression levels of regulators of inner root sheath and hair shaft differentiation in *iRhom2^Uncv/Uncv^* follicles

**Figure 6:**
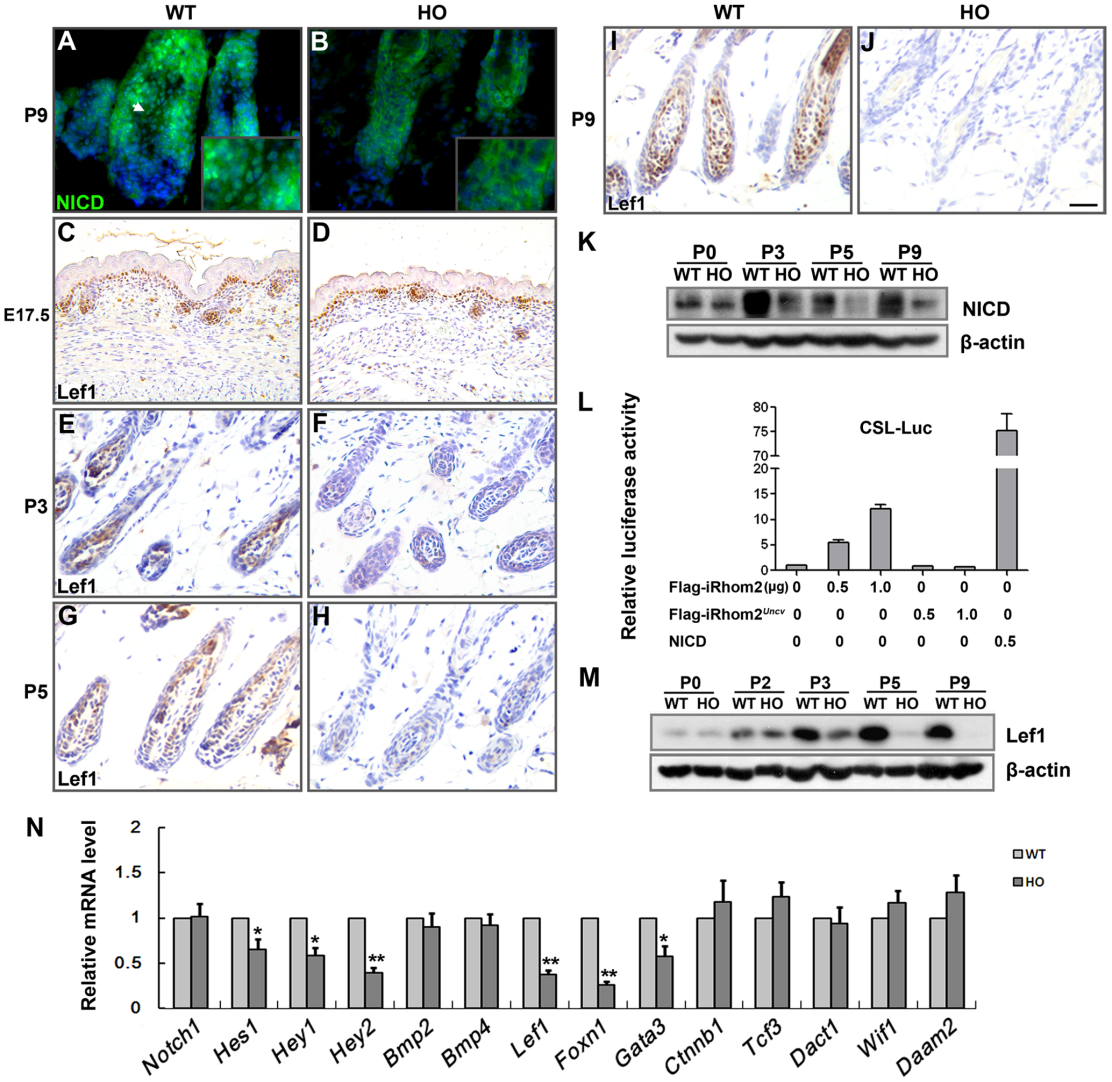
Expression levels of regulators of inner root sheath and hair shaft differentiation in *iRhom2^Uncv/Uncv^* follicles. (A, B) Immunofluorescence staining of NICD in the dorsal skin of P9 wild-type and age-matched *iRhom2^Uncv/Uncv^* mice. (C–J) Immunohistochemistry for Lef1 in the dorsal skin of E17.5, P0, P3 and P9 wild-type and *iRhom2^Uncv/Uncv^* mice. (K) Western blot analysis of NICD protein expression in the dorsal skin of wild-type and *iRhom2^Uncv/Uncv^* mice at P0, P3, P5 and P9. (L) Notch signaling transcriptional activity affected by Flag-iRhom2 or Flag-iRhom2*^Uncv^* mutation in HeLa cells. NICD serves as a positive control. (M) Western blot analysis of Lef1 protein expression in the dorsal skin of wild-type and *iRhom2^Uncv/Uncv^* mice at P0, P2, P3, P5 and P9. (N) Real-time PCR analysis of *Notch1*, *Hes1*, *Hey1*, *Hey2*, *Bmp2*, *Bmp4*, *Lef1*, *Foxn1*, *Gata3*, *Ctnnb1*, *Tcf3*, *Dact1*, *Wif1* and *Daam2* mRNA expression in the dorsal skin of wild-type and *iRhom2^Uncv/Uncv^* mice at P5. n = 3, *P<0.05, **P<0.01. Scale bars: (A, B), 12.5 µm; (C, D), 50 µm; (E–J), 25 µm.

Notch1 and BMP signaling play critical roles in the differentiation of the hair shaft and the IRS [12], [13], [15], [27]. Wnt/β-catenin signaling controls the differentiation of matrix cells along the hair shaft lineage [14]. TACE participates in the activation of the Notch pathway [28]–[31]. The expression of Notch intracellular domain (NICD) was dramatically reduced in *iRhom2^Uncv/Uncv^* mouse hair follicles from P3-P9 (Fig. 6A, B and K); however, the expression of *Notch1* mRNA was not significantly different from the wild-type (Fig. 6N), indicating that the post-translational maturation of the Notch1 protein was inhibited in *iRhom2^Uncv/Uncv^* mouse skin. The Notch1 target genes *Hes1*, *Hey1* and *Hey2* [27] were also downregulated in *iRhom2^Uncv/Uncv^* mouse skin (Fig. 6N). To determine whether iRhom2 affects Notch signaling transcriptional activity, HeLa cells were co-transfected with Notch-dependent CSL luciferase reporter (CSL-Luc) and increasing amounts of Flag-iRhom2 or Flag-iRhom2*^Uncv^* mutation. As shown in Fig. 6L, Notch transcriptional activation activated by iRhom2 in a dose-dependent manner, but iRhom2*^Uncv^* was not able to activate Notch transcriptional activation. It was reported that expression of Lef1, the Wnt/β-catenin pathway transcriptional effector, could be regulated by Notch1 [32], [33]. The expression of Lef1 was similar at E17.5 (Fig. 6C, D and M), dramatically reduced at P3 and P5 (Fig. 6E–H and M), and absent at P9 in *iRhom2^Uncv/Uncv^* mouse follicles compared to wild-type follicles (Fig. 6I, J and M). The expression of *Foxn1*, *Gata3* and *Hoxc13*, which are implicated in hair follicle differetionation [34], were dramatically reduced in *iRhom2^Uncv/Uncv^* mouse follicles (Fig. 6N, Figure E and F in S4 Fig.). The nuclear localization of β-catenin, which complexes with Lef1 to activate the transcription of Wnt target genes, was normal in *iRhom2^Uncv/Uncv^* mice (Figure A and B in S4 Fig.). Other genes involved in the Wnt pathway, including *Ctnnb1* (β-catenin), *Tcf3*, *Dact1*, *Wif1* and *Daam2* mRNA, were normally expressed in the dorsal skin of wild-type and *iRhom2^Uncv/Uncv^* mice at P5 (Fig. 6N). The levels of phospho-Smad1/5/8 were comparable between *iRhom2^Uncv/Uncv^* mouse follicles and the wild-type (Figure C and D in S4 Fig.), as were the expression levels of *Bmp2* and *Bmp4* mRNA (Fig. 6N). These results indicated that iRhom2 is involved in hair follicle differentiation by controlling the Notch and Wnt/β-catenin signaling pathways.

## Discussion

In this study, we discovered that iRhom2 regulate murine hair follicle differentiation by specifically modulating Notch1 and Wnt signaling pathway which maybe mediated by TACE.

Our study demonstrated that the *iRhom2^Uncv/Uncv^* mouse has hair differentiation abnormalities and displays a hairless phenotype in a BALB/c genetic background. The *iRhom2^Uncv/Uncv^* mouse showed decreased hair matrix proliferation and could not differentiated into IRS and hair shaft. In P9, the hair follicles of wild-type and mutant mice were in mid-anagen and had descended deep into the subcutis. However, the hair matrix of mutant follicles were less proliferative than that of wildtype. Comparable and few apoptotic cells were detected in both wildtype and *iRhom2^Uncv/Uncv^* hair matrix of P9, implying that mutant mice did not entered into catagen stage prematurely. Further examination showed that hair follicle cycling of mutant mice was normal. So, the marked shrinkage of the hair follicle matrix in mutant mice is not due to premature catagen development. The *iRhom2^Uncv/Uncv^* hair matrix failed to differentiate into IRS and hair shaft which resulted in the hairless phenotype of *iRhom2^Uncv/Uncv^* mouse. However, the hair follicle phenotype was not reported in *iRhom2* knockout C57BL/6 mouse [3], [5]. We noticed that *cub*/*cub* mice display a hairless or a wavy-coated phenotype depending on the modifier gene *mcub* [35]. When homozygous for the recessive *mcub* allele, *cub*/*cub* mice appear hairless. A single copy of the dominant *Mcub* allele confers a full, curly coat to *cub*/*cub* mice. The hairless phenotype in *cub*/*cub*, *mcub*/*mcub* mice resemble the *Uncv/Uncv* mice. The mapping regions of *cub* and *Uncv* cover a large overlapping region including the *iRhom2* gene. We speculate that *cub* and *Uncv* are the same gene, *iRhom2*. The modifier gene, *Mcub* or *mcub*, maybe affect hair follicle phenotype of *iRhom2* knockout mouse.

iRhom2 is necessary for the maturation of TACE. The *Adam17* knockout mice displayed a disorganized distribution and structure of hair follicles [36]. Consistent with phenotype in hair follicle of *Adam17* knockout mice, the *iRhom2^Uncv/Uncv^* mice showed irregularly positioned and oriented hair follicles with abnormal structure. *Adam17* conditional knockout mice induced by *K14-Cre* showed delayed hair outgrowth, shortened and disorganized hair follicles, abnormal epidermal proliferation and there was a dramatically increased infiltration of inflammatory macrophages [37]. *iRhom2^Uncv/Uncv^* mice showed defects in hair development and macrophages infiltration (Figure A, B and C in S5 Fig.) resembling those in *Adam17* conditional knockout mice. There are some disparity between *iRhom2^Uncv/Uncv^* mice and *Adam17* knockout mice. The majority of Adam17 knockout mice died between E17.5 and the first day after birth, those few mice that survived for several weeks had 20 to 40% body weights loss than those of littermates [36], while *iRhom2^Uncv/Uncv^* mice could survive up to 12 months and had 20% body weights loss at 4 weeks after birth. It was reported that there is some redundancy between them iRhom1 and iRhom2 [38], this provides some explanation *iRhom2^Uncv/Uncv^* mice do not show the several defects seen in *Adam17* knockouts. We noticed that *Adam17* knockouts have perturbed hair coats and curly vibrissae [36], however, *iRhom2^Uncv/Uncv^* mice have hairless phenotype. This discrimination implies that iRhom2 has additional physiologically substrates other than *Adam17*.

iRhom2 is required for controlling the activation of TACE-dependent shedding events [6]. Consistently, iRhom2*^Uncv^* cannot induce the maturation of TACE in vitro, and mature TACE was also significantly reduced in *iRhom2^Uncv/Uncv^* mouse skin. Furthermore, iRhom2*^Uncv^* does not affect the maturation of TACE induced by wild-type iRhom2. These findings indicated that iRhom2*^Uncv^* is a loss of function with respect to the maturation of TACE. However, the deleted region in the iRhom2*^Uncv^* mutant is not necessary for the degradation of EGF. So, the *iRhom2^Uncv^* mutant is not a simple loss of function mutation, which selectivity affects the client protein.

Our results indicated that the deleted region in the *iRhom2^Uncv^* is necessary for the maturation of TACE, which is required for the normal processing of Notch [28], [29], [39]. The Notch precursor protein is cleaved by furin to produce a bipartite heterodimeric molecule. Notch ligand-receptor interactions induce S2 and S3 proteolytic cleavage. S2 cleavage within the extracellular domain is mediated by TACE. Subsequently, S3 cleavage by the γ-secretase releases NICD, which translocates to the nucleus [39], [40]. We demonstrated that the *iRhom2^Uncv^* mutation affects TACE activity and inhibits the activation of Notch1. It was reported that Notch and WNT signaling exists cross-talk [32], [33], [41]. Moreover, the Notch intracellular domain can function as a coactivator for Lef1 and regulator for expression of Lef1 [32], [41]. We observed that the expression of NICD and Lef1 were dramatically reduced from P3, which is the initial stage of hair follicle differentiation and the numbers of hair matrix cells were comparable between the wild-type and the *iRhom2^Uncv/Uncv^* mice, indicating that the loss of NICD and Lef1 is not due to loss of the matrix cells. Therefore, the reduction in NICD and Lef1 expression in the *iRhom2^Uncv/Uncv^* mouse hair matrix appeared before the change in hair bulb morphology, which resulted in disorders of follicle differentiation. This study identifies that iRhom2 regulates hair shaft and IRS differentiation by specifically modulating Notch1 and Wnt signaling pathway which maybe mediated by TACE.

## Materials and Methods

### Mouse strains and genotyping

*Uncv* mice were maintained in a BALB/c background in a specific pathogen-free environment. *Uncv* mice were bred in a heterozygous mating heterozygous format. The genotyping primers were *iRhom2* fwd, 5′- CACAGCCCAGTGGTTTGGGGTCA -3′, and *iRhom2* rev, 5′- GAGGGCGGCGGCTGCCTGAAAGCT -3′. In wild-type mice, *iRhom2* was amplified by standard PCR to yield a 469 bp fragment. In *Uncv/Uncv* mutant mice, *iRhom2* was amplified to yield a 160 bp fragment. Wild-type mice littermates were used as controls. All animal studies were approved by the Review Board of the Institute of Radiation Medicine, Beijing, China.

### Plasmid construction

Full-length and mutant *iRhom2^Uncv^* were individually cloned into the pcDNA3.0-Flag vector using the primers *iRhom2* fwd, 5′-cccaagcttatggcctcagctgacaagaatggcagcaacctccca-3′, and *iRhom2* rev, 5′-ccggaattcttagtgtagcacctggtctagctcg-3′. Myc-tagged mouse EGF in pcDNA3.1 plasmid was a kind gift from Dr. Matthew Freeman [2].

### Cell culture, transfection and immunoprecipitation

293T cells were cultured in Dulbecco's modified Eagle's medium (DMEM) supplemented with 10% fetal bovine serum and 2 mM L-glutamine at 37°C in a humidified incubator with 5% CO_2_. iRhom2 plasmids and the pcDNA3.0-Flag vector were transfected into 293T cells using Lipofectamine 2000 (Invitrogen, Carlsbad, CA, USA). The immunoprecipitation was performed as described previously [42].

### Histological analysis, immunohistochemistry and immunofluorescence

Dorsal skin tissue samples were fixed in 4% paraformaldehyde at 4°C overnight, embedded in paraffin, sectioned at 4 µm and stained with hematoxylin and eosin for histological analysis. Immunohistochemistry and immunofluorescence was performed as described previously [43], [44]. For the iRhom2 antibody-blocking experiment, iRhom2 antibody was pre-mixed with iRhom2 antibody (N-terminus) blocking peptides (Abgent) before incubation with the sections.

### Terminal deoxyribonucleotidyl transferase-mediated dUTP nick-end labeling assay

The terminal deoxynucleotidyl transferase dUTP nick-end labeling (TUNEL) assay was performed on 4-µm-thick sections of dorsal skin using the ApopTag Peroxidase In Situ Apoptosis Detection Kit (Roche) following the manufacturer's directions.

### RNA isolation and real-time PCR

Total RNA was isolated from the dorsal skin using TRIzol reagent (Life Technologies) following the manufacturer's protocol, and cDNA was then prepared using the Prime Script RT reagent kit (TaKaRa) according to the manufacturer's instructions with oligo-dT primers. SYBR Premix Ex *Taq* (TaKaRa) was used for real-time quantification, and gene expression was normalized to *GAPDH* using the ΔΔcycle threshold method. Primer sequences are available upon request. To assess the *iRhom2* expression separately in the epidermis and dermis, dorsal skin was incubated in 0.25% trypsin at 4°C for 24 to 48 hours; the epidermis was removed as soon as it could be separated from the dermis as an intact sheet.

### Luciferase assays

HeLa cells were transfected with Notch-dependent CSL luciferase reporter containing CSL binding sites, Renilla luciferase reporter, iRhom2 and iRhom2*^Uncv^* plasmids. Renilla luciferase was used as a transfection control and signals are given as fold Firefly/Renilla corrected for background. 48 h after transfection, luciferase-reporters activity was measured. Datas are representative of at least three independent experiments.

### Statistical analysis

All results are presented as the means *±* SE. All statistical analyses were performed using the SPSS software. The significance of the differences between groups was determined using Student's *t*-test; *P*<0.05 was considered significant.

## Supporting Information

S1 Fig **Genotyping of wild-type,** ***iRhom2^Uncv/+^*** **and** ***iRhom2^Uncv/Uncv^*** **mice.** (A) All homozygous *Uncv/Uncv* mice are hairless, and all heterozygotes (HE) are sparsely coated. (B) The PCR genotyping of *iRhom2* revealed a 469 bp wild-type PCR product and a 160 bp mutant product. Heterozygous mice produced both the 469 bp and 160 bp products.
(TIF)

S2 Fig **Expression of iRhom2 in mouse skin.** (A) Real-time PCR analysis of *iRhom2*, *iRhom2#* (The PCR primers were located in the deletion region of *iRhom2^Uncv^*), mRNA expression in the dorsal skin of wild-type and *iRhom2^Uncv/Uncv^* mice at P5. n = 3, **P<0.01. (B, C) Immunofluorescence staining of iRhom2 at P9 mouse dorsal skin from wild-type mice. (D, E) Immunofluorescence staining of iRhom2 and TACE in the dorsal skin of P9 wild-type and *iRhom2^Uncv/Uncv^* mice. Scale bars: (B–E), 12.5 µm.
(TIF)

S3 Fig **Histology of the dorsal skin from wild-type and** ***iRhom2^Uncv/Uncv^*** **mice.** (A–F) Histology of the dorsal skin from wild-type and *iRhom2^Uncv/Uncv^* mice at P18, P22 and P32, respectively. Scale bars: (A–F), 100 µm.
(TIF)

S4 Fig **Expression levels of β-catenin, phospho-Smad1/5/8 and Hoxc13 in** ***iRhom2^Uncv/Uncv^*** **follicles.** (A–F) Immunofluorescence staining of β-catenin, phospho-Smad1/5/8 and Hoxc13 in the dorsal skin of P9 wild-type and *iRhom2^Uncv/Uncv^* mice. Scale bars: (A–F), 12.5 µm.
(TIF)

S5 Fig **Excessive macrophages infiltration in** ***iRhom2^Uncv/Uncv^*** **mice skin.** (A, B) Immunohistochemistry staining of skin with anti-F4/80 antibodies to detect macrophages in wild-type and *iRhom2^Uncv/Uncv^* mice at P5. (C) Number of F4/80-positive cells per 20× field in the dermis of P9; n = 5, **P<0.01. Scale bars: (A, B), 25 µm.
(TIF)
