# Supplementary figures and images for: iRhom2 Mutation Leads to Aberrant Hair Follicle Differentiation in Mice
Source: PLoS One. 2014 Dec 29;9(12):e115114. doi: 10.1371/journal.pone.0115114 (PMC4278852; doi:10.1371/journal.pone.0115114)

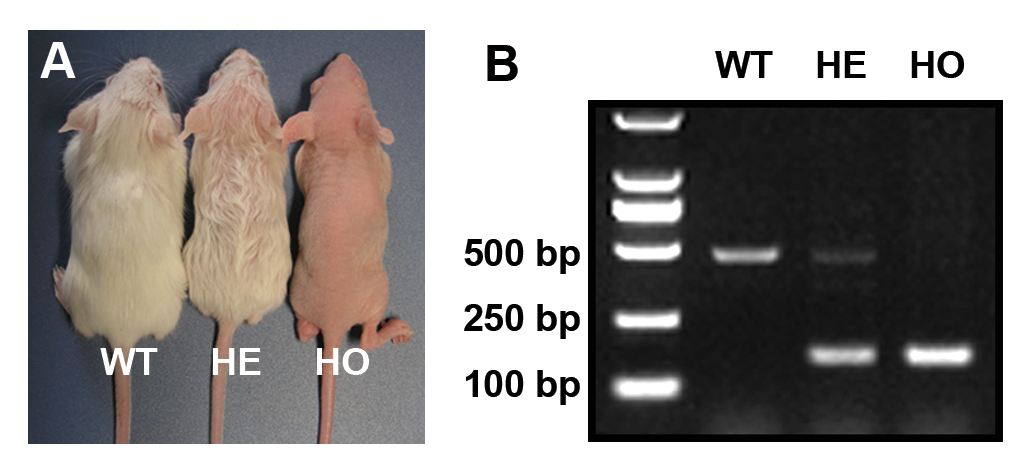

Supplement: S1 Fig — Genotyping of wild-type, iRhom2Uncv/+ and iRhom2Uncv/Uncv mice. (A) All homozygous Uncv/Uncv mice are hairless, and all heterozygotes (HE) are sparsely coated. (B) The PCR genotyping of iRhom2 revealed a 469 bp wild-type PCR product and a 160 bp mutant product. Heterozygous mice produced both the 469 bp and 160 bp products. (TIF) [file pone.0115114.s001.tif]

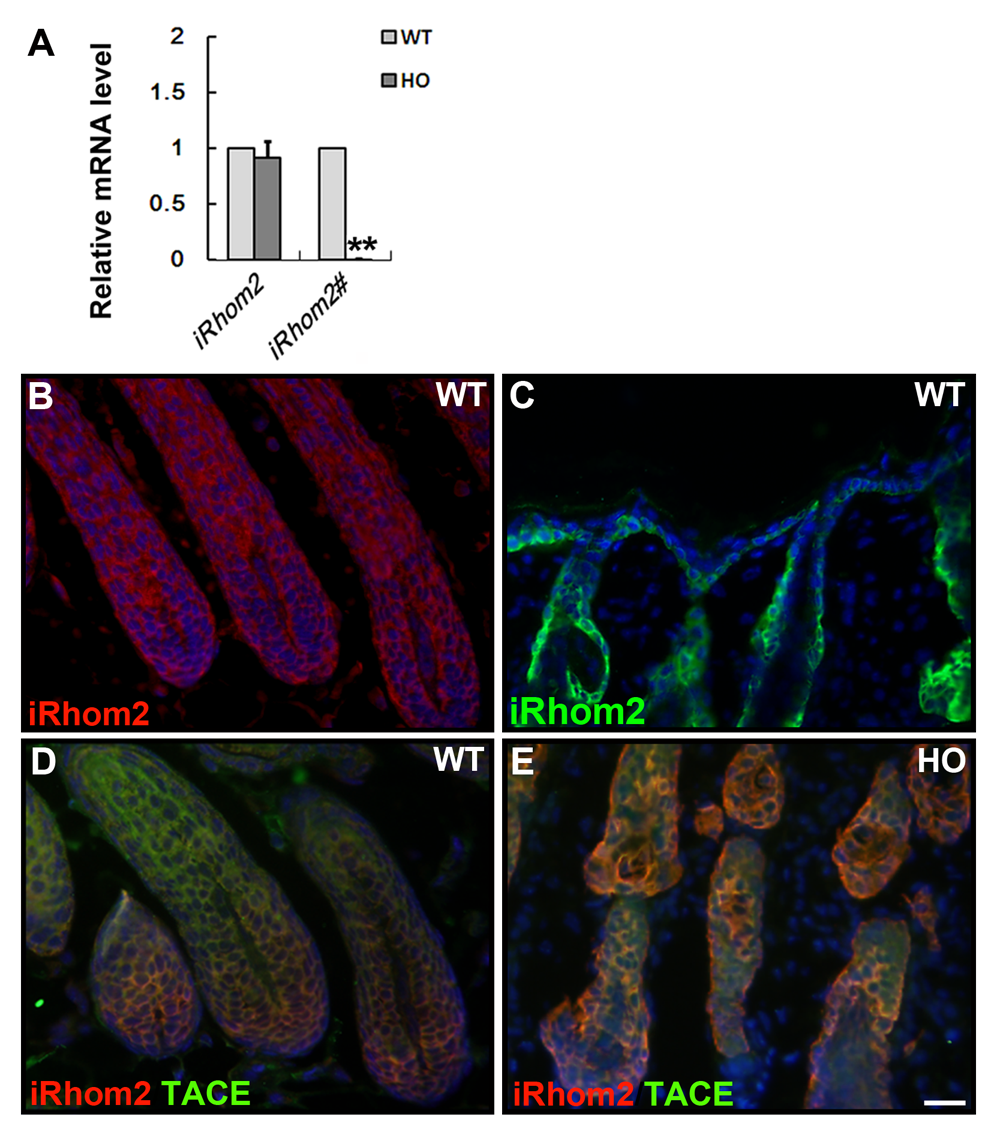

Supplement: S2 Fig — Expression of iRhom2 in mouse skin. (A) Real-time PCR analysis of iRhom2, iRhom2# (The PCR primers were located in the deletion region of iRhom2Uncv), mRNA expression in the dorsal skin of wild-type and iRhom2Uncv/Uncv mice at P5. n = 3, **P<0.01. (B, C) Immunofluorescence staining of iRhom2 at P9 mouse dorsal skin from wild-type mice. (D, E) Immunofluorescence staining of iRhom2 and TACE in the dorsal skin of P9 wild-type and iRhom2Uncv/Uncv mice. Scale bars: (B–E), 12.5 µm. (TIF) [file pone.0115114.s002.tif]

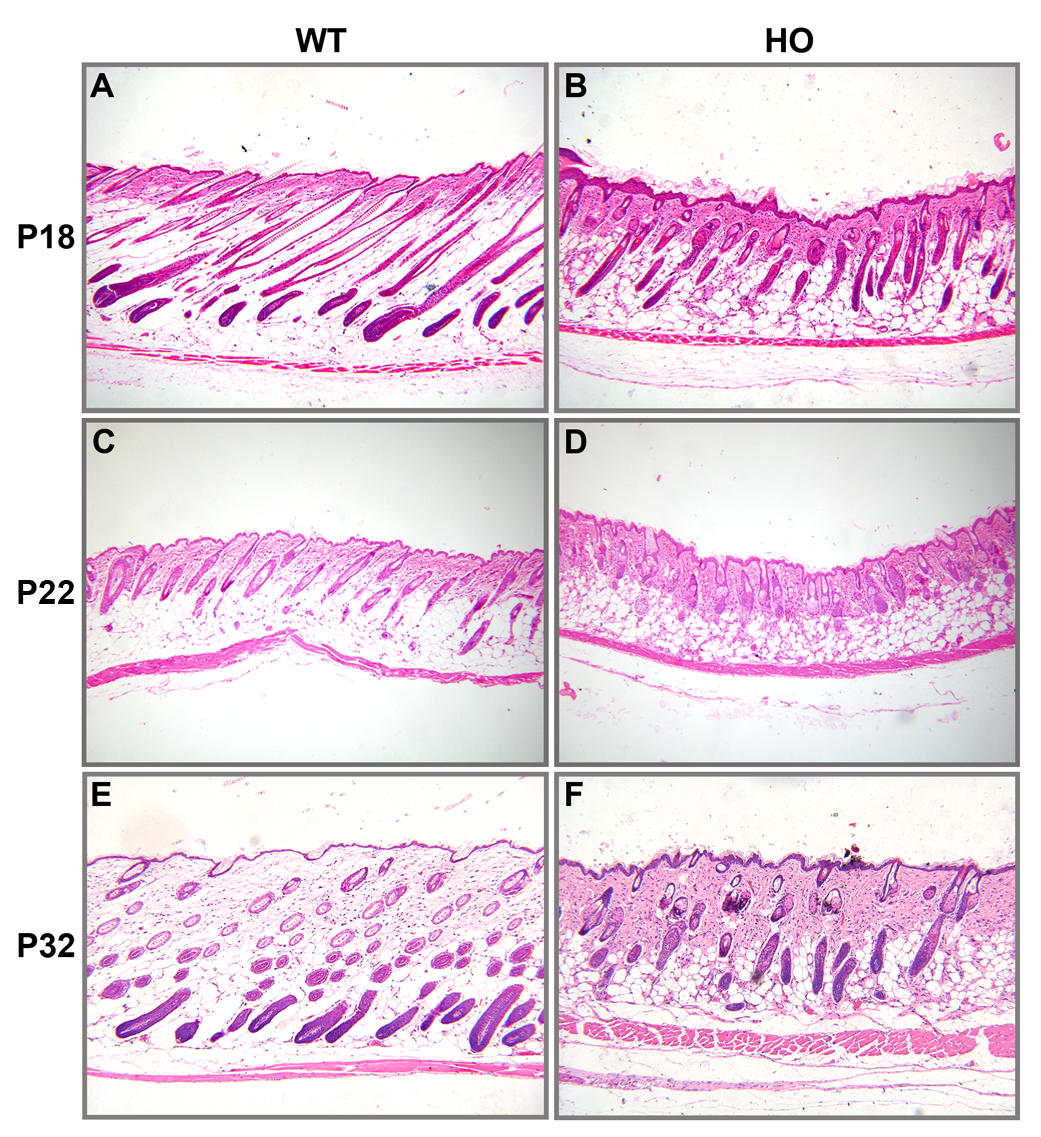

Supplement: S3 Fig — Histology of the dorsal skin from wild-type and iRhom2Uncv/Uncv mice. (A–F) Histology of the dorsal skin from wild-type and iRhom2Uncv/Uncv mice at P18, P22 and P32, respectively. Scale bars: (A–F), 100 µm. (TIF) [file pone.0115114.s003.tif]

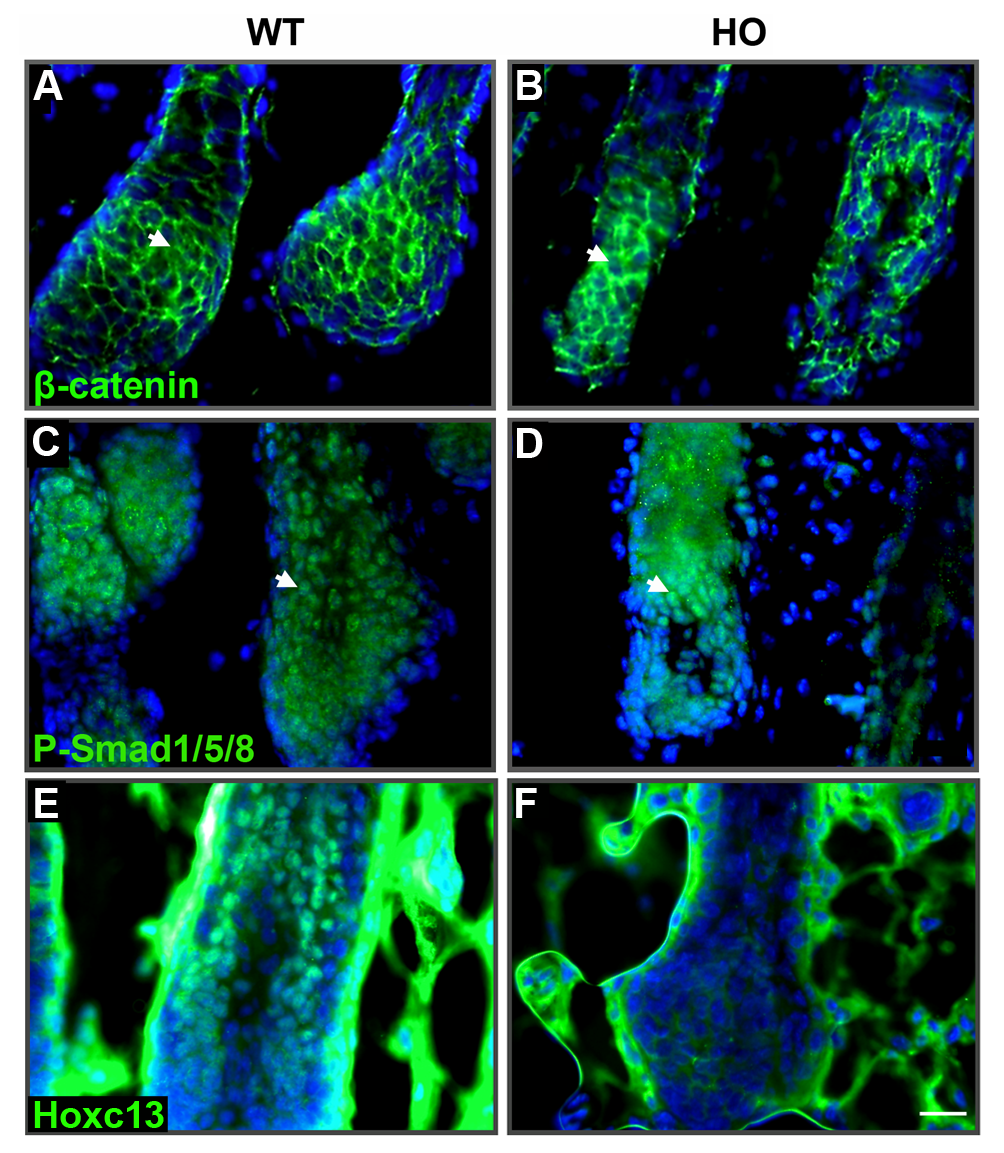

Supplement: S4 Fig — Expression levels of β-catenin, phospho-Smad1/5/8 and Hoxc13 in iRhom2Uncv/Uncv follicles. (A–F) Immunofluorescence staining of β-catenin, phospho-Smad1/5/8 and Hoxc13 in the dorsal skin of P9 wild-type and iRhom2Uncv/Uncv mice. Scale bars: (A–F), 12.5 µm. (TIF) [file pone.0115114.s004.tif]

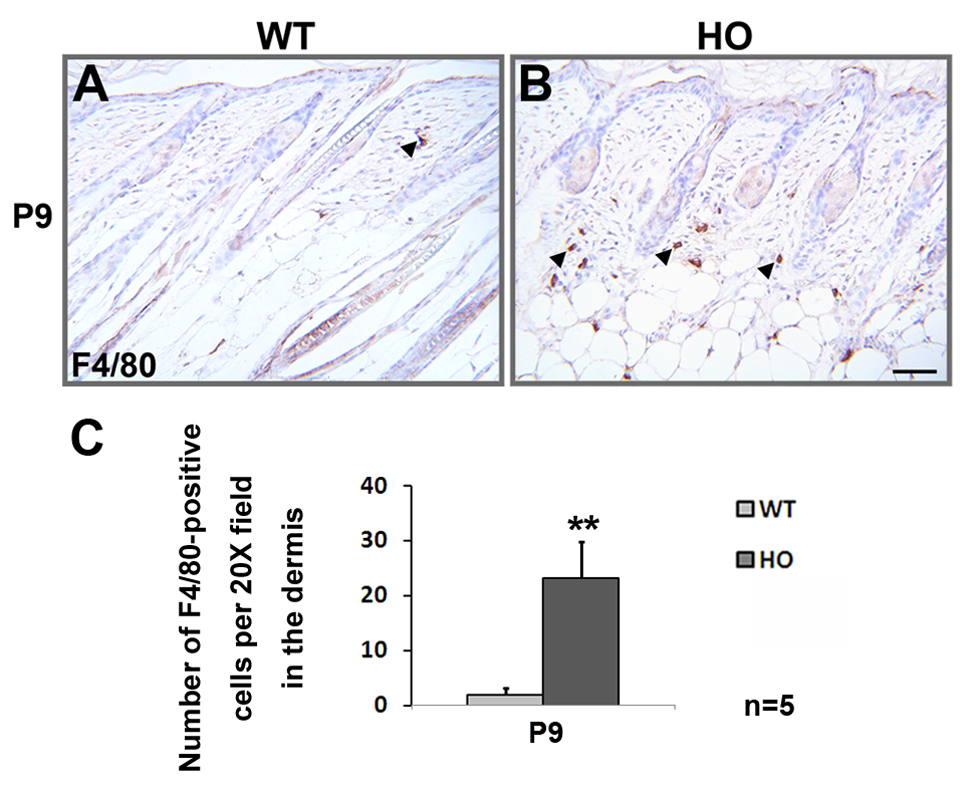

Supplement: S5 Fig — Excessive macrophages infiltration in iRhom2Uncv/Uncv mice skin. (A, B) Immunohistochemistry staining of skin with anti-F4/80 antibodies to detect macrophages in wild-type and iRhom2Uncv/Uncv mice at P5. (C) Number of F4/80-positive cells per 20× field in the dermis of P9; n = 5, **P<0.01. Scale bars: (A, B), 25 µm. (TIF) [file pone.0115114.s005.tif]
